# Supplementary material for: Assessing risks of dengue, chikungunya and Zika transmission associated to Aedes albopictus in Chania, Greece, 2017–2018
Source: PLoS Negl Trop Dis. 2025 Dec 1;19(12):e0013785. doi: 10.1371/journal.pntd.0013785 (PMC12700377; doi:10.1371/journal.pntd.0013785)
Supplement: S1 Text — (DOCX) [file pntd.0013785.s001.docx]

S1 Text

**Assessing risks of dengue, chikungunya and Zika transmission associated to *Aedes albopictus* in Chania, Greece, 2017-2018**

Sk Shahid Nadim ^1,2,*^, Francesco Menegale ^1,*^, Mattia Manica ^1^, Alexander R. Kaye ^3,4^, Georgios Balatsos ^5^, Marina Bisia ^5^, Verena Pichler ^6^, Piero Poletti ^1^, Stefano Merler ^1^, Alessandra della Torre ^7^, Robin N. Thompson ^8^, Antonios Michaelakis ^5^, Giorgio Guzzetta ^1, #^

^1^ Centre for Health Emergencies, Bruno Kessler Foundation, Trento, Italy

^2^ Department of Mathematics, SRM University-AP, Amaravati, Andhra Pradesh, India

^3^ Mathematics Institute, University of Warwick, Coventry, UK

^4^ Zeeman Institute for Systems Biology and Infectious Disease Epidemiology Research (SBIDER), University of Warwick, Coventry, UK

^5^ Laboratory of Insects & Parasites of Medical Importance, Benaki Phytopathological Institute, Athens, Greece

^6^ Saint Camillus International University of Health and Medical Sciences, Rome, Italy

^7^ Department of Public Health and Infectious Diseases, “Sapienza” University, Rome, Italy

^8^ Mathematical Institute, University of Oxford, Oxford, UK

^*^ Co-first authors

^#^ Corresponding author: Giorgio Guzzetta – guzzetta@fbk.eu

Table of Contents

[**Model equations for mosquito populations** 2](#_Toc213079879)

[**Calibration procedure** 3](#_Toc213079880)

[**Comparison of observed and modelled captures** 4](#_Toc213079881)

[**Estimation of reproduction numbers** 7](#_Toc213079882)

[**Epidemic risks** 11](#_Toc213079883)

[**Sensitivity analysis** 20](#_Toc213079884)

[**References** 23](#_Toc213079885)

### **Model equations for mosquito populations**

We adapted the mathematical model by Guzzetta et al [1] to simulate the life cycle dynamics of *Ae. albopictus*, covering all life stages: eggs, larvae, pupae, and adult females. This model integrates established temperature-dependent mortality rates and transition rates between developmental stages, from eggs to larvae, larvae to pupae, and pupae to adult mosquitoes, providing a comprehensive framework to study population dynamics. The model equations are expressed as follows

$$\frac{dE}{dt}=n_{E}d_{V}V-\left( m_{E}+d_{E} \right)E$$

$$\frac{dL}{dt}=d_{E}E-\left( m_{L}\left( 1+\frac{L}{a_{S}} \right)+d_{L} \right)L$$

$$\frac{dP}{dt}=d_{L}L-\left( m_{P}+d_{P} \right)P$$

$$\frac{dV}{dt}=\frac{1}{2}d_{P}P-\left( m_{V}+\alpha\right)V.$$

where E, L, P, and V denote the numbers of eggs, larvae, pupae, and female adult mosquitoes, respectively. Here, $n_{E}$ represents the average number of eggs laid per female adult during oviposition, while $d_{V}$ signifies the temperature-driven rate at which female adults deposit eggs [1]. The parameters $d_{i}$ (where i = E, L, P) denote temperature-dependent rates governing the progression from one developmental stage to the next [2]. Additionally, $m_{i}$ (with i = E, L, P, V) stands for temperature-dependent mortality rates for each stage of development [2]. The term $a_{S}$ represents larval overcrowding and $\alpha$ represents the capture rate for adult female mosquitoes to the site, where each site is equipped with a single trap during the capture process. We assume that the modeled population refers to a circular area centered around the trap with radius equal to the mosquito flight range assumed to be 250m [1,3].

**Table A.** Biological parameters for *Ae. albopictus*, $T$ denotes the temperature in Celsius.

| Biological parameters | | | |
| --- | --- | --- | --- |
| Parameters | Description | Value/Formula | Ref |
| $\boldsymbol{d}_{\boldsymbol{E}}\boldsymbol{(T)}$ | Developmental rates correspond to egg hatching | $\frac{1}{d_{e1}-d_{e2} {exp\{-((T-20)/d_{e3})}^{2}}$ | 1,2 |
| $\boldsymbol{d}_{\boldsymbol{L}}\boldsymbol{(T)}$ | Developmental rates correspond to larvae | $\frac{1}{d_{l1} T^{2}+d_{l2}T +d_{l3}}$ | 1,2 |
| $\boldsymbol{d}_{\boldsymbol{P}}\boldsymbol{(T)}$ | Developmental rates correspond to pupation | $\frac{1}{d_{p1} T^{2}+d_{p2}T +d_{p3}}$ | 1,2 |
| $\boldsymbol{d}_{\boldsymbol{V}}\boldsymbol{(T)}$ | Egg deposition rate | $\frac{1}{d_{v1} T^{2}+d_{v2}T +d_{v3}}$ | 1,2 |
| $\boldsymbol{\mu}_{\boldsymbol{E}}\boldsymbol{(T)}$ | Mortality rates for eggs | $\mu_{e1}-\mu_{e2} {exp\{-((T-25)/\mu_{e3})}^{6}\}$ | 1,2 |
| $\boldsymbol{\mu}_{\boldsymbol{L}}\boldsymbol{(T)}$ | Mortality rates for larvae | $\mu_{l1}+\mu_{l2} exp(T-\mu_{l3})$ | 1,2 |
| $\boldsymbol{\mu}_{\boldsymbol{P}}\boldsymbol{(T)}$ | Mortality rates for pupae | $\mu_{p1}+\mu_{p2} exp(T-\mu_{p3})$ | 1,2 |
| $\boldsymbol{\mu}_{\boldsymbol{V}}\boldsymbol{(T)}$ | Mortality rates for adult mosquitoes | $If T< {15}^{o} C$  $\mu_{V} = \frac{1}{1.1+exp\left( -4.04+0.576*T \right)} +0.12$  $If {15}^{o} C \leq T < {26.3}^{o} C$  $\mu_{V} = 0.000339 *T^{2} - 0.0189 * T +0.336$  $If T\geq{26.3}^{o} C$  $\mu_{V} = \frac{1}{1.065+exp\left( 32.2+0.092*T \right)} +0.0747$ | 3 |
| $\boldsymbol{\beta(T)}$ | Biting rate (${day}^{-1}$) | $\beta_{1}*(\beta_{2}*T+\beta_{3})$ | 3 |
| $\boldsymbol{\phi}$ | Human host preference | $0.6$ | 3 |

**Table B.** Developmental rates, mortality rates and biting rates of *Ae. Albopictus*.

| Development cycle | | | | | | | |
| --- | --- | --- | --- | --- | --- | --- | --- |
| $\boldsymbol{d}_{\boldsymbol{e}\boldsymbol{1}}$ | 6.9 | $d_{l1}$ | 0.12 | $d_{p1}$ | 0.027 | $d_{v1}$ | 0.046 |
| $\boldsymbol{d}_{\boldsymbol{e}\boldsymbol{2}}$ | 4.0 | $d_{l2}$ | -6.6 | $d_{p2}$ | -1.7 | $d_{v2}$ | -2.77 |
| $\boldsymbol{d}_{\boldsymbol{e}\boldsymbol{3}}$ | 4.1 | $d_{l3}$ | 98 | $d_{p3}$ | 27.7 | $d_{v3}$ | 45.3 |
| Mortality rates | | | | | | | |
| $\boldsymbol{\mu}_{\boldsymbol{e}\boldsymbol{1}}$ | 506 | $\mu_{l1}$ | 0.029 | $\mu_{p1}$ | 0.021 | $\beta_{1}$ | 0.5 |
| $\boldsymbol{\mu}_{\boldsymbol{e}\boldsymbol{2}}$ | 506 | $\mu_{l2}$ | 858 | $\mu_{p2}$ | 37 | $\beta_{2}$ | 0.0043 |
| $\boldsymbol{\mu}_{\boldsymbol{e}\boldsymbol{3}}$ | 27.3 | $\mu_{l3}$ | 43.4 | $\mu_{p3}$ | 36.8 | $\beta_{3}$ | 0.0943 |

**Temperature over time**

Fig A reports the temperatures for the study site for 2017 and 2018.

**Fig A.** Observed temperatures over time for 2017 and 2018

### **Calibration procedure**

We performed the model calibration using data from May 1, 2017, to December 31, 2017, and from April 1, 2018, to December 31, 2018, initialising all state variables at zero, except for an initial number of eggs of 10,000. The temperature-dependent functional forms for the rates, as estimated in earlier studies [1,2] using experimental data [4], are provided in Table A along with their associated parameter in Table B.

We integrated Poisson likelihoods derived from the observed data across the two seasons. This statistical method allowed us to compare and adjust our model's predictions against the actual number of captures recorded in our experiments. The 8 site-specific larval overcrowding $a_{S}$ and the capture rate $\alpha$, were the free model parameters, for which we assumed uniform prior distributions across a broad range of values. To determine the posterior distributions, which reflect updated beliefs about the parameters given observed data, we employed a Monte Carlo Markov Chain (MCMC) method. Specifically, we used a random-walk Metropolis-Hastings sampling technique, where proposals for new parameter values were generated from normal (Gaussian) jump distributions centered around the current parameter values. The predicted cumulative number of females captured during each session was fitted to the observed data using the Poisson likelihood, which is defined as follows:

$$L\left( M,p | \alpha,a_{s} \right)=\prod_{s}^{S} \prod_{j}^{N_{s}} \frac{e^{-p_{sj}(\alpha,a_{s})}{p_{sj}(\alpha,a_{s})}^{M_{sj}}}{M_{sj}!}$$

$M_{sj}$ is the observed number of captured female mosquitoes during capture session $j$ at study site $s$, $p_{sj}$ is the model-predicted number of captured females by capture session and study site, depending on free model parameters $\alpha$ and $a_{s}$, $S$ is the number of study sites ($S$ = 8), $N_{s}$ is the number of capture sessions for site $s$.

To ensure the MCMC procedure converged to the true posterior distributions, we ran 50,000 iterations of sampling with a burn-in-period of 15,000. We visually inspected the remaining iterations to confirm convergence and obtained 175 parameter sets by sampling from the resulting posterior distributions. Table C presents the posterior means and 95% confidence intervals (CI) of estimates for the free model parameters i.e. the capture rate and the larval overcrowding for both seasons, as determined by MCMC.

**Table C.** Estimates of model parameters from the MCMC calibration procedure.

| **Parameter (unit of measure)** | | **2017** | **2018** |
| --- | --- | --- | --- |
| Larval overcrowding $a_{S}$  (adimensional) | Mean | $2.582$ | $3.624$ |
|  | 95% CI | $2.387-2.867$ | $3.482-3.757$ |
| Capture rate $\alpha$(day^-1^) | Mean | $7.485\times{10}^{-4}$ | |
|  | 95% CI | $7.307\times{10}^{-4}- 7.919\times{10}^{-4}$ | |

### **Comparison of observed and modelled captures**

The Fig B displays the positions of the eight BG-Sentinel 2 traps near the Chania airport. Figs C and D compare the observed and the modeled numbers of captured adult females for 2017 and 2018 respectively for the eight traps, showing their average values and 95% credible intervals. Despite the inherent variability in the observed numbers due to different times and seasons, the modeled estimates generally follow the observed seasonal pattern. Figs E and F show the model estimates of daily female adult mosquito density for 2017 and 2018 respectively for the eight traps. For all epidemiological analyses, we select mosquito abundance estimates from the trap with highest abundance for each year, i.e., BG 6 for 2017 and BG 3 for 2018.


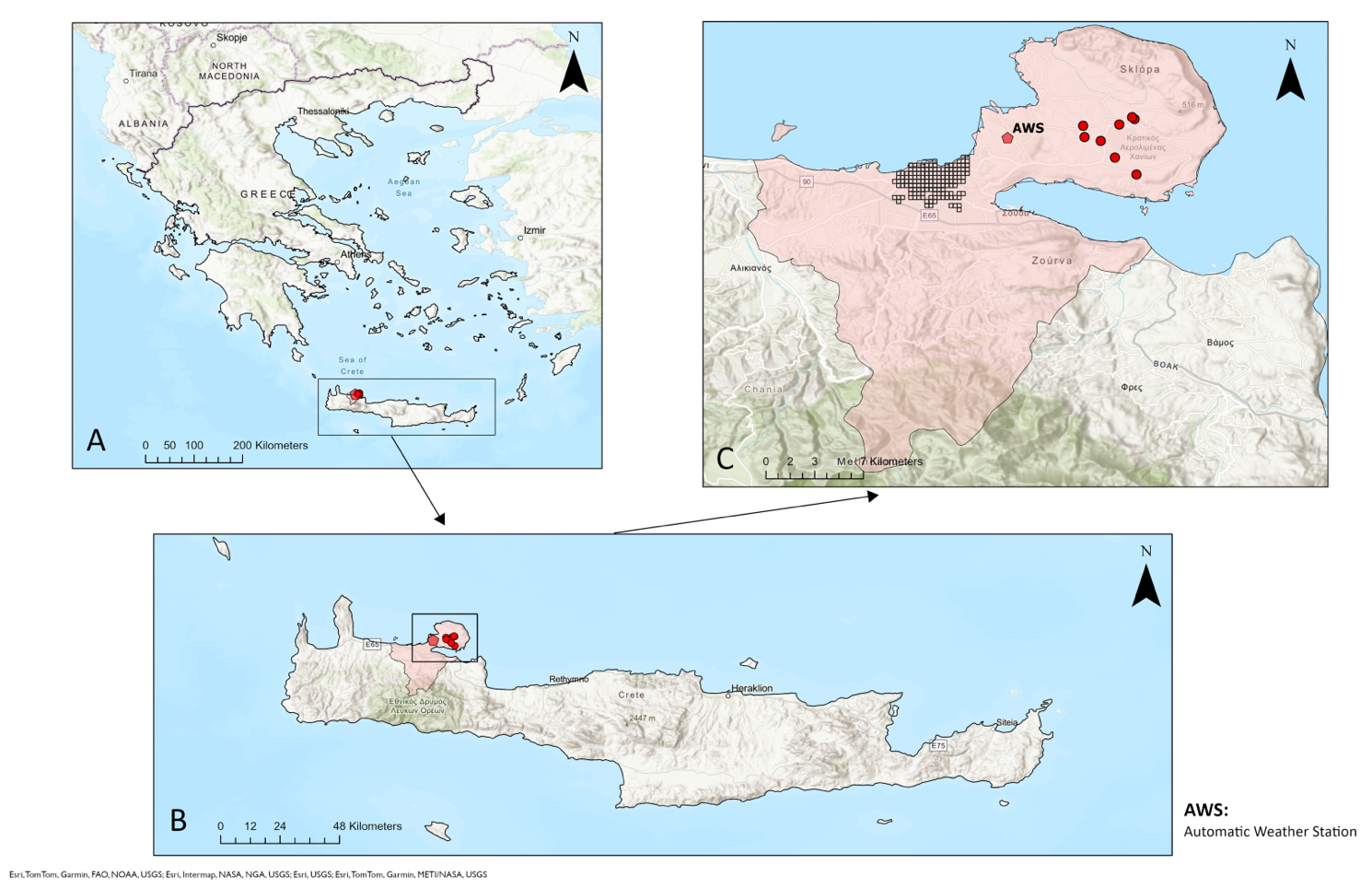


**Fig B**. Geography of the study area. A) The island of Crete within Greece; B) the municipality of Chania within the island of Crete; C) pink area: administrative borders of the municipality of Chania (pink); red dots: position of the eight BG-Sentinel 2 traps; red pentagon: Akrotiri Automated Weather Station (AWS); grid: the residential area of the town of Chania where arboviral risks were estimated, divided in 147 square cells of side 250m. Background map layer was created with ArcGIS Pro (3.0.0), using the World Topographic Map available from Esri (Marathon Data, GR) under Esri Master License Agreement (https://www.arcgis.com/home/item.html?id=30e5fe3149c34df1ba922e6f5bbf808f). The administrative boundaries for the municipality of Chania were retrieved from [5], using functions available in the ggmap package for R version 4.0.0 [6], together with a private API key.

**Fig C.** Comparison between observed and modeled captures over time for eight BG-Sentinel 2 traps. Adult females captured, depicted as red squares, and the modeled numbers, shown in black, including their average values and 95% confidence intervals for 2017.

**Fig D.** Comparison between observed and modeled captures over time for eight BG-Sentinel 2 traps. Adult females captured, depicted as red squares, and the modeled numbers, shown in black, including their average values and 95% confidence intervals for 2018.

**Fig E.** Model estimates of daily female adult mosquito density for 2017 for eight BG-Sentinel 2 traps. Solid lines: mean; shaded areas: 95% CrI.

**Fig F.** Model estimates of daily female adult mosquito density for 2017 for eight BG-Sentinel 2 traps. Solid lines: mean; shaded areas: 95% CrI.

### **Estimation of reproduction numbers**

We evaluated the potential risk of chikungunya and dengue outbreaks that arise from the introduction of a single infectious individual into the study area, assuming no control measures are in place. For this analysis, we considered a classical host-vector SEI-SEIR transmission dynamic model. In this model, female adult mosquitoes become infected after biting an infectious human host. They then undergo an extrinsic incubation period before becoming capable of transmitting the infection to other hosts, remaining infectious for the duration of their life. On the other hand, when an infectious mosquito bites a susceptible human, the human enters an intrinsic incubation period during which the virus incubates within them. Following this latent phase, the human becomes infectious, during which they can transmit the infection to other mosquitoes when bitten. The human becomes permanently immune after clearance of the infection. Within a population of completely susceptible mosquitoes and human hosts, the average number of mosquitoes infected by a single infectious human host is denoted by $R_{HV}\left( t \right)$. Similarly, within a population of completely susceptible mosquitoes and human hosts, the average number of human host infected by a single infectious mosquito is denoted by $R_{VH}(t)$ (see Main text).

The basic reproduction number is the average number of secondary human infections resulting from the introduction of a single infected host in a population of completely susceptible mosquitoes and hosts. This may be calculated as the product of these two variables:

$$R_{0}\left( t \right) = R_{HV}R_{VH}={\beta(t)}^{2}\phi^{2}\frac{N_{V}(t)}{N_{H}}\frac{\chi_{V}(t)\chi_{H}(t)}{\mu_{V}(t)\gamma}\frac{\omega_{V}(t)}{\omega_{V}(t)+\mu_{V}(t)}$$

For dengue, the role of asymptomatic individuals in transmission remains debated, with evidence from cohort studies suggesting that asymptomatic dengue infections are characterized by lower viral titers and distinct viremia kinetics compared to symptomatic cases, possibly resulting in lower infectiousness [7]. Modeling studies have adopted different assumptions about the relative transmissibility of symptomatic and asymptomatic cases [3,8]. To acknowledge the potentially higher contribution of symptomatic individuals to transmission, we included a multiplicative factor $p$ into the equation for $R_{HV}$, whose range reflects different assumptions about the relative transmissibility of symptomatic and asymptomatic individuals. This range captures scenarios where symptomatic individuals represent a smaller proportion of infections - such as in endemic countries, where they typically account for around 20% - yet contribute more to transmission than asymptomatic cases, as well as others where symptomatic individuals are the majority (such as in a dengue outbreak in the Lombardy region, Italy; [9]) or where all individuals, asymptomatic and symptomatic, contribute equally to the transmission ($p=1$). It is worth noting that the factor $p$, under the assumption that asymptomatic individuals do not transmit, coincides with the fraction of symptomatic individuals. However, $p$ should not be interpreted strictly as the fraction of symptomatic individuals, but rather as a proxy for the overall contribution of all infected individuals (asymptomatic and symptomatic) in a context where symptomatic individuals may be more infectious.

All model parameters used for the presented analysis are summarized in Table D.

**Table D**. Epidemiological parameters of mosquitoes for chikungunya, dengue, and Zika. $T$ denotes the temperature in Celsius.

| Epidemiologial parameters | | | | |
| --- | --- | --- | --- | --- |
| Disease | Parameters | Description | Value/Formula | Ref |
| DENV | 1/$\gamma$ | Infectious period in humans (days) | 2 - 5 | 10,11 |
|  | $\chi_{V}$ | Mosquito susceptibility to infections | ${4.39 * {10}^{-4} * T * (T-3.62) * (36.82-T)}^{1/2}$ | 12 |
|  | $\chi_{H}$ | Human susceptibility to infections | ${7.35 * {10}^{-4} * T * (T-15.84) * (36.40-T)}^{1/2}$ | 12 |
|  | $p$ | Proportion of symptomatic infections | $0.25-1$ | 3,8 |
|  | $\omega_{V}$ | Mosquito extrinsic incubation rate (${day}^{-1}$) | ${1.09 * {10}^{-4} * T * (T-10.39) * (43.05-T)}^{1/2}$ | 12 |
| CHIKV | 1/$\gamma$ | Infectious period in humans (days) | 2 - 7 | 2,3,13,14 |
|  | $\chi_{V}$ | Mosquito susceptibility to infections | 70% - 100% | 2 |
|  | $\chi_{H}$ | Human susceptibility to infections | 50% - 80% | 2 |
|  | 1/$\omega_{V}$ | Mosquito extrinsic incubation period (days) | 2 - 3 | 2 |
| ZIKAV | 1/$\gamma$ | Infectious period in humans (days) | 4 - 7 | 15,16 |
|  | $\chi_{V}$ | Mosquito susceptibility to infections | 6.65% - 10% | 16,17 |
|  | $\chi_{H}$ | Human susceptibility to infections | 1% - 100% | 16,17 |
|  | 1/$\omega_{V}$ | Mosquito extrinsic incubation period (days) | 7 - 14 | 16 |

The estimates are given for 147 cells with a human population of more than 10 individuals per hectare, at a spatial scale of 250 m x 250 m. The human population density is showed in Fig G.

**Fig G.** Human population density of Chania with more than 10 individuals per hectare, at a spatial scale of 250 m x 250 m. Map was created using QGIS software version 3.30.2. Background map layer was obtained from OpenStreetMap (<https://www.openstreetmap.org>) and is made available under the Open Database License (<http://opendatacommons.org/licenses/odbl/1.0/>). Any rights in individual contents of the database are licensed under the Database Contents License (<http://opendatacommons.org/licenses/dbcl/1.0/>).

Fig H displays the spatial distribution of the average monthly basic reproduction number for Dengue virus (DENV) in Chania for the months of June to October in 2017 and 2018.


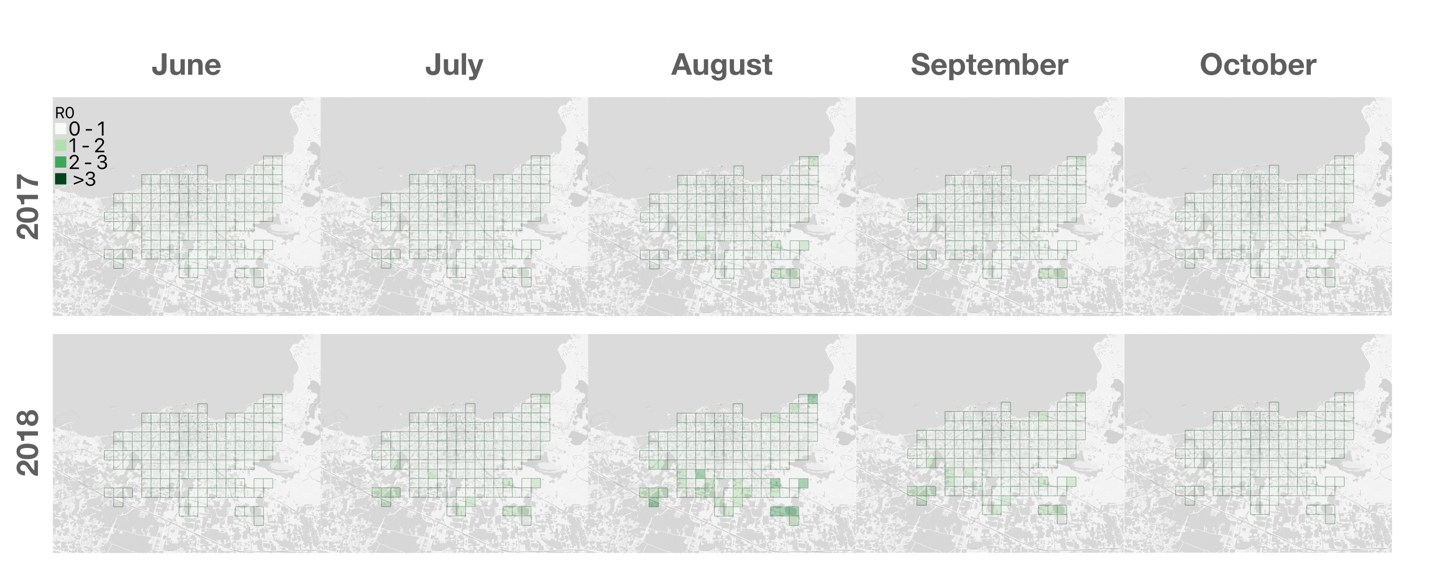


**Fig H**. Estimated temporal dynamics of the reproduction number, R_0_, for dengue virus in Chania for 2017 and 2018 respectively. Estimates are provided at a spatial scale of 250 m x 250 m, focusing on 147 cells with a human density over 10 individuals per hectare. Maps were created using QGIS software version 3.30.2. Background map layer was obtained from OpenStreetMap (<https://www.openstreetmap.org>) and is made available under the Open Database License (<http://opendatacommons.org/licenses/odbl/1.0/>). Any rights in individual contents of the database are licensed under the Database Contents License (<http://opendatacommons.org/licenses/dbcl/1.0/>).

Fig I displays the spatial distribution of the average monthly basic reproduction number for Chikungunya virus (CHIKV) in Chania for the months of June to October in 2017 and 2018.


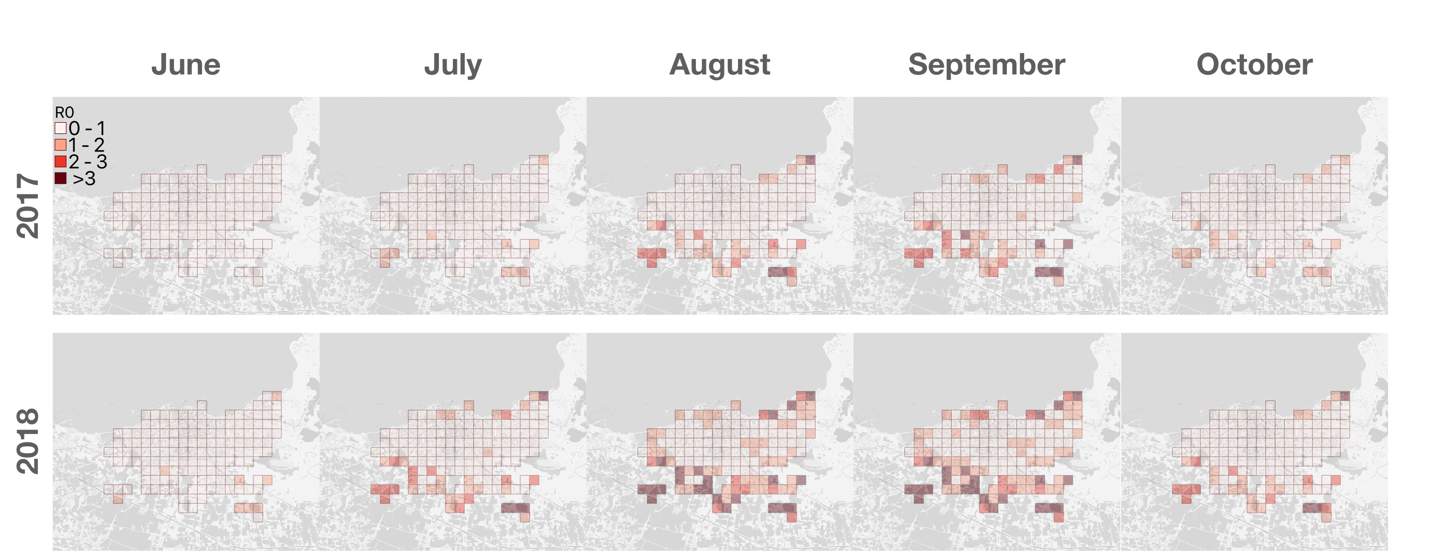


**Fig I.** Estimated temporal dynamics of the reproduction number, R_0_, for Chikungunya virus in Chania for 2017 and 2018 respectively. Estimates are provided at a spatial scale of 250 m x 250 m, focusing on 147 cells with a human density over 10 individuals per hectare. Maps were created using QGIS software version 3.30.2. Background map layer was obtained from OpenStreetMap (<https://www.openstreetmap.org>) and is made available under the Open Database License (<http://opendatacommons.org/licenses/odbl/1.0/>). Any rights in individual contents of the database are licensed under the Database Contents License (<http://opendatacommons.org/licenses/dbcl/1.0/>).

### **Epidemic risks**

Even with a reproduction number R_0_ above 1, transmission may fade out due to stochastic effects. We compute the probability of a major outbreak to quantify the chances that an initial imported infection will lead to sustained onward transmission. To this aim, we used two alternative estimates. The first, termed Instantaneous Epidemic Risk (IER), is computed in closed form based on the classical SEIR-SEI model formulation [18], described in the Main text.

Here, the IER varies over time due to changes in N_V_, as well as temperature-dependent parameters in both R_HV_ and R_VH_ (see Main text). Fig J illustrates the relationship between season length, defined as the number of days with IER exceeding zero and average IER computed across the season for dengue and chikungunya in 2017 and 2018. There are noticeable variations in the patterns of both diseases, but they both exhibit an increase in the average risk as the epidemic season grows longer.


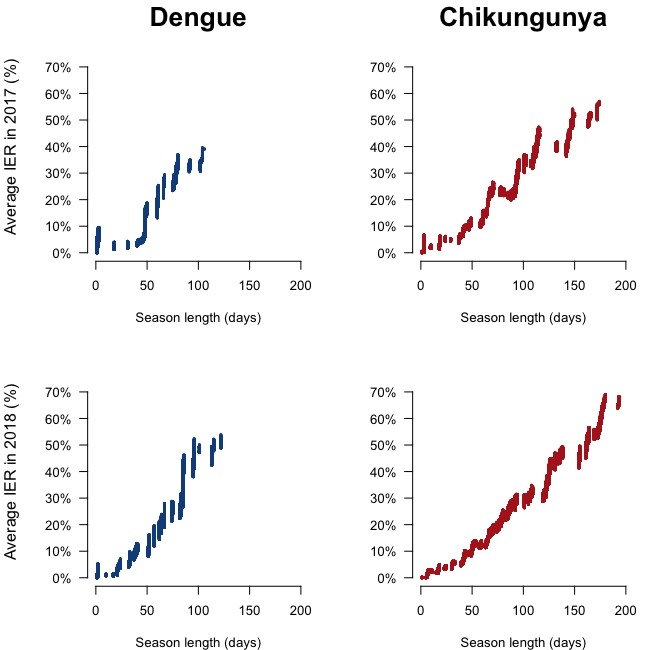


**Fig J.** Scatter plot between the average IER computed over the epidemic season and season length for dengue (left panels) and chikungunya (right panels) in 2017 (top row) and 2018 (bottom row).

In Figs K and L we show the relationship between the estimated average season lengths and IER across the 147 cells and the corresponding human population density. A negative correlation is observed, but it changes over the years and cannot be obviously reconducted to a simple analytical function.

**Fig K.** Scatter plot between estimated average season lengths and population density over the 147 cells for dengue (left panels) and chikungunya (right panels) in 2017 (top row) and 2018 (bottom row).

**Fig L.** Scatter plot between estimated average IER and population density over the 147 cells for dengue (left panels) and chikungunya (right panels) in 2017 (top row) and 2018 (bottom row).

The Instantaneous Epidemic Risk (IER) assumes the virus spreads at a constant rate once it enters a population, but this is only an approximation of the real outbreak risk. A more accurate estimation can be achieved through computational simulations. In these simulations, a single infectious individual is introduced into the population, and mosquito abundances as well as epidemiological parameters are updated dynamically over time. For each cell in the municipality, we ran 100,000 simulations of a stochastic model analogous to the SEIR-SEI model, using an adaption of the Gillespie algorithm for systems with temporally varying parameters (see Supplementary). The 100,000 simulations were made up of 100 simulations for each of the 1,000 samples from the prior distribution of literature-estimated parameters and from the posterior distributions of the mosquito density time-series. We then determined the risk of an outbreak for each cell by counting the proportion of the 100,000 simulations that resulted in 10 infections or more. This estimate of risk is referred to as the "Threshold Epidemic Risk" (TER). The spatial distribution of TER and IER for dengue and chikungunya for case introductions occurring on the first day of the month between June and October 2017 is presented in Fig M.


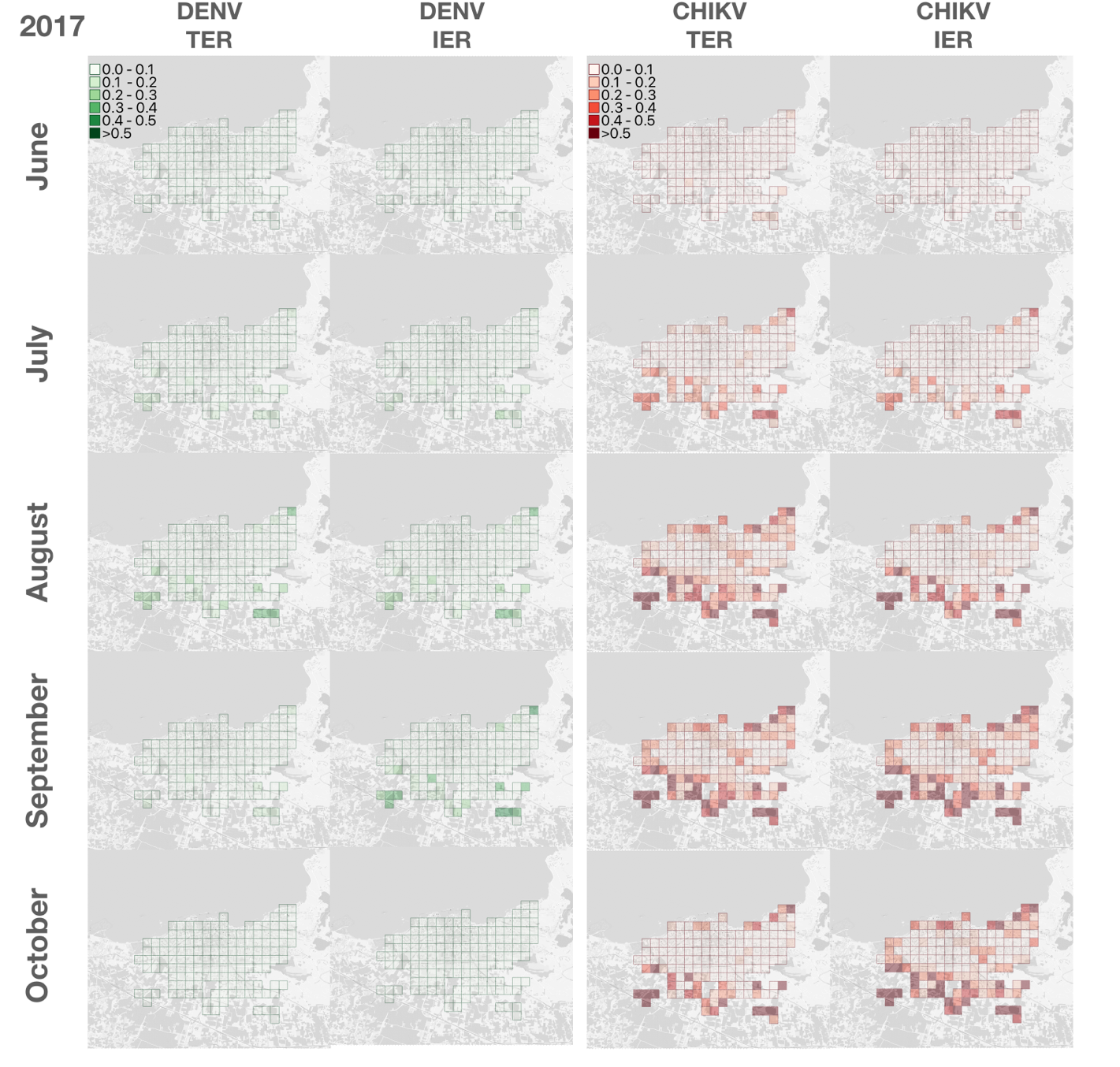


**Fig M.** Estimated Threshold Epidemic Risk (TER) and Instantaneous Epidemic Risk (IER) for dengue and chikungunya for 2017. Estimates are provided at a spatial scale of 250 m × 250 m, focusing on 147 cells with a human density over 10 individuals per hectare. Maps were created using QGIS software version 3.30.2. Background map layer was obtained from OpenStreetMap (<https://www.openstreetmap.org>) and is made available under the Open Database License (<http://opendatacommons.org/licenses/odbl/1.0/>). Any rights in individual contents of the database are licensed under the Database Contents License (<http://opendatacommons.org/licenses/dbcl/1.0/>).

The spatial distribution of TER and IER for dengue and chikungunya for case introductions occurring on the first day of the month between June and October 2018 is presented in Fig N.


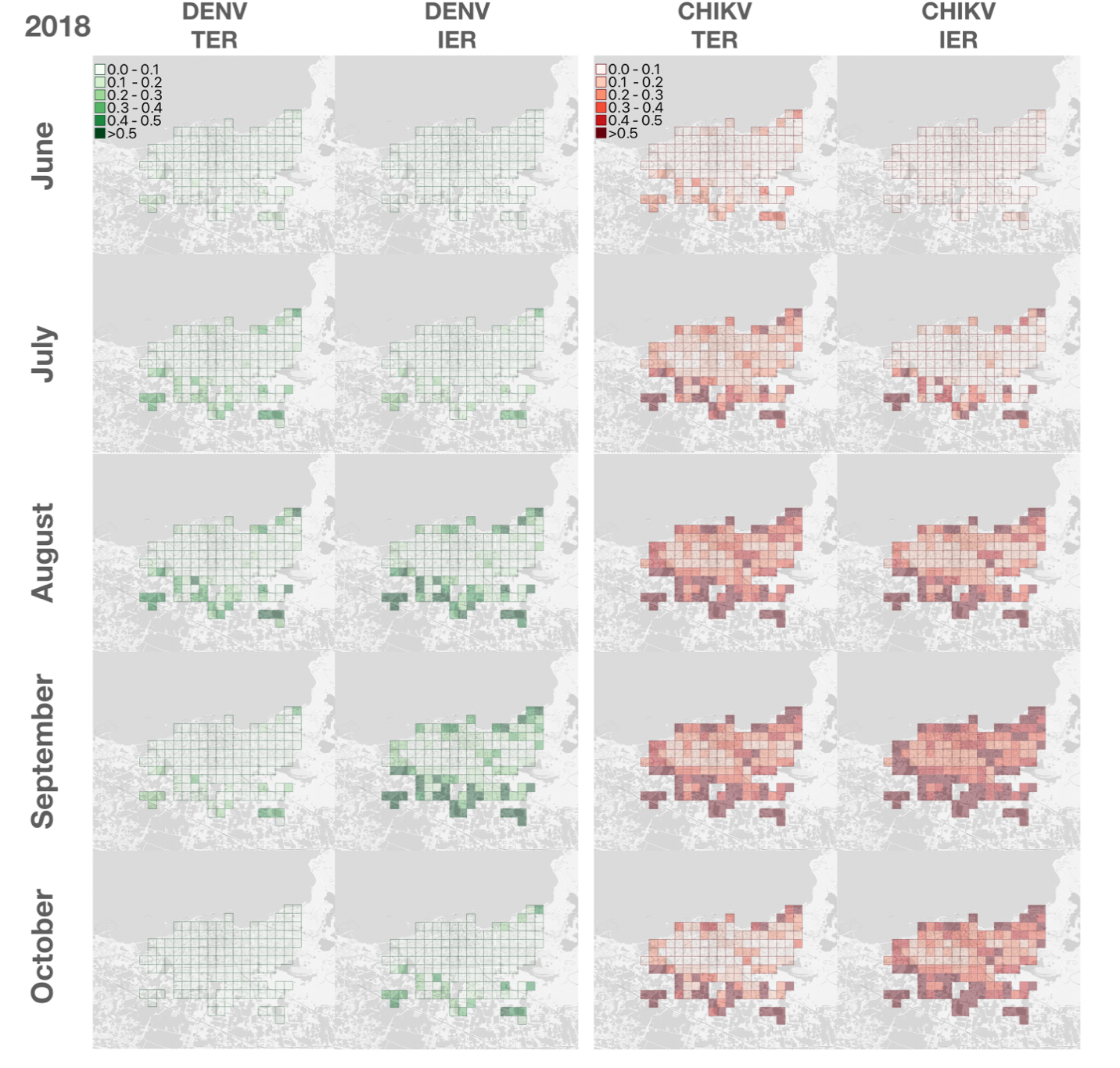


**Fig N.** Estimated Threshold Epidemic Risk (TER) and Instantaneous Epidemic Risk (IER) for Dengue and Chikungunya for 2018. Estimates are provided at a spatial scale of 250 m × 250 m, focusing on 147 cells with a human density over 10 individuals per hectare. Maps were created using QGIS software version 3.30.2. Background map layer was obtained from OpenStreetMap (<https://www.openstreetmap.org>) and is made available under the Open Database License (<http://opendatacommons.org/licenses/odbl/1.0/>). Any rights in individual contents of the database are licensed under the Database Contents License (<http://opendatacommons.org/licenses/dbcl/1.0/>).

We fitted a linear regression model to estimate the relationship between how the month of introduction of the infected cases and host population density impacted the diﬀerence between Threshold Epidemic Risk (TER) and Instantaneous Epidemic Risk (IER) estimates. We applied a frequentist approach for the estimation of model parameters. We considered the diﬀerence between TER and IER as the dependent variable and the month of introduction and the density of human hosts in each cell of the municipality as the independent variable. We express diﬀerence between TER and IER in fraction, to avoid risk of misinterpretation of the difference TER-IER as a relative difference. We considered the month as a categorical variable and used August as reference in the regression model. The adopted model equation is given by:

$$\mu_{i} = \alpha+ \beta1 {June}_{i} + \beta2 {July}_{i}+ \beta3 {September}_{i} + \beta4 {October}_{i} + \beta5 {Density}_{i}$$

Subscript i = 1..N, represent each observation in the two years in each one of the 147 cell in the 5 months of introduction considered. Since TER and IER are not defined for negative values, in order to avoid biases in their quantitative difference, we filtered only cases where both TER and IER are non-zero in the main analysis. In Figs O-R, we show sensitivity analyses where we also include cases where either the TER, the IER, or both estimates are zero.

**Fig O.** Relationship between TER and IER estimates of the probability of dengue and chikungunya outbreaks, assuming the introduction of a viremic index case on the first day of June, July, August, September and October of 2017 and 2018. Left: distributions of the probability of outbreaks, obtained by pooling together estimates from both years across cells, excluding those where the TER is zero; horizontal bar: mean; boxes: interquartile range; vertical lines: 95 percentile range. Right: coefficients of a linear regression model for the difference between TER and IER. Points represent the mean difference, and the vertical bands represent the 95% confidence interval.

**Fig P.** Relationship between TER and IER estimates of the probability of dengue and chikungunya outbreaks, assuming the introduction of a viremic index case on the first day of June, July, August, September and October of 2017 and 2018. Left: distributions of the probability of outbreaks, obtained by pooling together estimates from both years across cells, excluding those where the IER is zero; horizontal bar: mean; boxes: interquartile range; vertical lines: 95 percentile range. Right: coefficients of a linear regression model for the difference between TER and IER. Points represent the mean difference, and the vertical bands represent the 95% confidence interval.

**Fig Q.** Relationship between TER and IER estimates of the probability of dengue and chikungunya outbreaks, assuming the introduction of a viremic index case on the first day of June, July, August, September and October of 2017 and 2018. Left: distributions of the probability of outbreaks, obtained by pooling together estimates from both years across cells, excluding those where both the TER and IER are zero; horizontal bar: mean; boxes: interquartile range; vertical lines: 95 percentile range. Right: coefficients of a linear regression model for the difference between TER and IER. Points represent the mean difference, and the vertical bands represent the 95% confidence interval.

**Fig R.** Relationship between TER and IER estimates of the probability of dengue and chikungunya outbreaks, assuming the introduction of a viremic index case on the first day of June, July, August, September and October of 2017 and 2018. Left: distributions of the probability of outbreaks, obtained by pooling together estimates from both years across cells and selecting all values for both TER and IER; horizontal bar: mean; boxes: interquartile range; vertical lines: 95 percentile range. Right: coefficients of a linear regression model for the difference between TER and IER. Points represent the mean difference, and the vertical bands represent the 95% confidence interval.

### **Sensitivity analysis**

In the main analysis, we considered epidemiological parameters for *Ae. albopictus* to be constant with respect to temperature, in absence of published data on their temperature dependence. Because the experimental temperature for the measurement of such parameters is on the upper side of the temperature range observed in Chania (generally around 28°C), we conducted a sensitivity analysis where we considered temperature-dependent functions of mosquito susceptibility, host susceptibility, and extrinsic incubation period for chikungunya, borrowed from the corresponding ones established for dengue, but rescaled in such a way to match the experimental value for chikungunya at the experimental temperature. If $f_{x}(T)$ is the temperature-dependent function observed for parameter $x$ for dengue, $G_{x}$ is the value of the constant chikungunya parameter and $T_{x}^{exp}$ is the temperature at which the experiment for $G_{x}$ was conducted, we considered a temperature-dependent function for chikungunya given by $g_{x}\left( T \right)= a\cdot f_{x}(T)$, where $a=\frac{f_{x}\left( T_{x}^{exp} \right)}{G_{x}}$. We verified that the resulting values remained in physically plausible ranges (i.e., all values were non-negative and susceptibility parameters never exceeded 100%).

Re-running chikungunya simulations under these assumptions resulted in qualitatively similar results as in the main analysis (Figs N-P), although the transmissibility in the later part of the season decreased more briskly. This resulted in shorter season lengths and lower risks for chikungunya compared to the main analysis, but still larger corresponding estimates for dengue.

**Fig S.** Comparison of estimated time-varying reproduction numbers with constant parameters (main analysis) and temperature-dependent mosquito susceptibility, host susceptibility, and extrinsic incubation period.

**Fig T.** Comparison of estimated distributions of the peak reproduction numbers with constant parameters (main analysis, left) and temperature-dependent mosquito susceptibility, host susceptibility, and extrinsic incubation period (right).

**Fig U.** Comparison of estimated time-varying season lengths (top panels) and average risk (bottom panels) with constant parameters (main analysis, left panels) and temperature-dependent mosquito susceptibility, host susceptibility, and extrinsic incubation period (right panels).

### **References**

1. Guzzetta G, Montarsi F, Baldacchino FA, Metz M, Capelli G, Rizzoli A, et al. Potential Risk of Dengue and Chikungunya Outbreaks in Northern Italy Based on a Population Model of Aedes albopictus (Diptera: Culicidae). PLoS Negl Trop Dis. 2016 June;10(6):e0004762.
2. Poletti P, Messeri G, Ajelli M, Vallorani R, Rizzo C, Merler S. Transmission Potential of Chikungunya Virus and Control Measures: The Case of Italy. PLOS ONE. 2011 May 3;6(5):e18860.
3. Zardini A, Menegale F, Gobbi A, Manica M, Guzzetta G, d’Andrea V, et al. Estimating the potential risk of transmission of arboviruses in the Americas and Europe: a modelling study. The Lancet Planetary Health. 2024 Jan 1;8(1):e30–40.
4. Delatte H, Gimonneau G, Triboire A, Fontenille D. Influence of Temperature on Immature Development, Survival, Longevity, Fecundity, and Gonotrophic Cycles of Aedes albopictus, Vector of Chikungunya and Dengue in the Indian Ocean. J Med Entomol. 2009 Jan 1;46(1):33–41.
5. Geoadata.gov.gr. Municipality boundaries (Kallikratis) - Όρια Δήμων (Καλλικράτης) [Internet]. 2025 [cited 2025 Oct 20]. Available from: <http://geodata.gov.gr/en/dataset/oria-demon-kallikrates/resource/6643d54a-f1af-4841-ad99-7a49a1d13650>
6. Kahle D, Wickham H, Jackson S, Korpela M. ggmap: Spatial Visualization with ggplot2 [Internet]. 2025 [cited 2025 Oct 20]. Available from: <https://cran.r-project.org/web/packages/ggmap/index.html>
7. Matangkasombut P, Manopwisedjaroen K, Pitabut N, Thaloengsok S, Suraamornkul S, Yingtaweesak T, et al. Dengue viremia kinetics in asymptomatic and symptomatic infection. Int J Infect Dis. 2020 Dec;101:90–7.
8. Ferguson NM, Rodríguez-Barraquer I, Dorigatti I, Mier-Y-Teran-Romero L, Laydon DJ, Cummings DAT. Benefits and risks of the Sanofi-Pasteur dengue vaccine: Modeling optimal deployment. Science. 2016 Sept 2;353(6303):1033–6.
9. Rovida F, Faccini M, Molina Granè C, Cassaniti I, Senatore S, Rossetti E, et al. The 2023 dengue outbreak in Lombardy, Italy: A one-health perspective. Travel Medicine and Infectious Disease*.* 2025 Mar 1;64:102795.
10. Brady OJ, Golding N, Pigott DM, Kraemer MUG, Messina JP, Reiner RC, et al. Global temperature constraints on Aedes aegypti and Ae. albopictus persistence and competence for dengue virus transmission. Parasit Vectors. 2014 July 22;7:338.
11. Marini G, Guzzetta G, Marques Toledo CA, Teixeira M, Rosà R, Merler S. Effectiveness of Ultra-Low Volume insecticide spraying to prevent dengue in a non-endemic metropolitan area of Brazil. PLoS Comput Biol. 2019 Mar;15(3):e1006831.
12. Mordecai EA, Cohen JM, Evans MV, Gudapati P, Johnson LR, Lippi CA, et al. Detecting the impact of temperature on transmission of Zika, dengue, and chikungunya using mechanistic models. PLoS Negl Trop Dis. 2017 Apr;11(4):e0005568.
13. Manore CA, Hickmann KS, Xu S, Wearing HJ, Hyman JM. Comparing dengue and chikungunya emergence and endemic transmission in A. aegypti and A. albopictus. Journal of Theoretical Biology. 2014 Sept 7;356:174–91.
14. Massad E, Ma S, Burattini MN, Tun Y, Coutinho FAB, Ang LW. The risk of chikungunya fever in a dengue-endemic area. J Travel Med. 2008;15(3):147–555.
15. Caminade C, Turner J, Metelmann S, Hesson JC, Blagrove MSC, Solomon T, et al. Global risk model for vector-borne transmission of Zika virus reveals the role of El Niño 2015. Proc Natl Acad Sci U S A. 2017 Jan 3;114(1):119–24.
16. Guzzetta G, Poletti P, Montarsi F, Baldacchino F, Capelli G, Rizzoli A, et al. Assessing the potential risk of Zika virus epidemics in temperate areas with established Aedes albopictus populations. Euro Surveill. 2016 Apr 14;21(15).
17. Di Luca M, Severini F, Toma L, Boccolini D, Romi R, Remoli ME, et al. Experimental studies of susceptibility of Italian Aedes albopictus to Zika virus. Euro Surveill. 2016 May 5;21(18).
18. Lloyd AL, Zhang J, Root AM. Stochasticity and heterogeneity in host–vector models. Journal of The Royal Society Interface. 2007 June 19;4(16):851–63.
