# Supplementary material for: Assessing risks of dengue, chikungunya and Zika transmission associated to Aedes albopictus in Chania, Greece, 2017–2018
Source: PLoS Negl Trop Dis. 2025 Dec 1;19(12):e0013785. doi: 10.1371/journal.pntd.0013785 (PMC12700377; doi:10.1371/journal.pntd.0013785)
Supplement: Attachment — Submitted filename: PlosNTDs_review_Aug2025_complete.docx [file pntd.0013785.s003.docx]

Summary

Dear editors,

This review is for the article “Assessing risks of dengue, chikungunya and Zika transmission associated to *Aedes albopictus* in Chania, Greece, 2017-2018.” The paper is original and interesting to read. I have written my review comments below, I hope the authors find them helpful.

My main comment is that the methods section needs expanding because it is often unclear what was performed without going back and forth to the Supplementary Material, and so I believe more methodological detail is needed in the main text.

Overall, the content of this paper meets the requirements from the journal: it is of interest to researchers and practitioners outside the field as well as in the field of NTDs.

I have split my comments into clarifications that are necessary and also more minor suggestions which I hope the authors find helpful.

Thank you to the authors for writing this interesting paper.

Kind regards,

Victoria Cox

Major points

Line 135. The free model parameters include the larval-overcrowding and the capture rate for the adult females. How was the larval-overcrowding calibrated? Was this using the ovitrap data? This is unclear and more explanation would be helpful.

Line 147. “considering mosquito populations associated to the trap with highest abundance over each year”. Does this mean that data from only one of the traps was used? It is unclear why this was done, please can you expand on the reasoning. How do the results change if the mosquito population is averaged over the traps instead? If I’ve understood correctly what data was used here, then this needs to be talked about in the discussion as a potential introduction of bias.

Line 145. Is R0(t) the R0 or is it Rt? Please clarify this in the text.

This work originally looked like a spatial model to me. Whereas I now see that the model was run independently for each of the 147 cells with no modelling of correlation across space. This is fine, but I think the distinction needs to be made clearer from the beginning of the article. Minor suggestion on this point - it could be useful to give an overall outbreak risk value for the whole municipality, assuming a constant averaged human population density first, and then describe the cell-specific results. Or have a graph of risk versus human population perhaps.

Similar to the point above, the only difference between the cells is the human population density. The mosquito population and climate are constant across the whole area and any spatial difference in R0 and outbreak risk is wholly dependent on the human population density. (1) Is it possible there could be large spatial differences in the mosquito population density across the municipality? It seems from the supplementary figures that even across the 8 traps there was some heterogeneity. Some more discussion is needed around how spatial differences in mosquito populations would impact the results. Could areas of higher human population density sustain a larger mosquito population carrying capacity? (2) Because so much of the results focusses on the differences in risk between the cells, more information/equations on how human population density is used to calculate the R0 and risk is needed in the main methods text.

In the Supplement it says that for dengue, it was assumed that only individuals displaying symptoms contribute to virus transmission. What is the justification for this?

Table S4 has mosquito susceptibility for ZIKV as a very low maximum ~10% whereas 70-100% for CHIKV which is likely why the authors see no risk for ZIKV outbreaks. Since it has such a big impact on the results, I think more discussion on these values would be useful. i.e. are the referenced % values representative of the literature? Is there any chance the ZIKV value could be higher and therefore the risk underestimated?

The data availability statement suggests that all data is available, however the mosquito capture data is only included in a figure and therefore not practically available. For reproducibility, this article would benefit from source code being made available for example through a GitHub repository, and data similarly made available externally or at least given in tables. If the mosquito data can’t be made available (I appreciate this is often not possible) then the data availability statement needs to be updated to express under what circumstances researchers could request access and from where.

Minor points

Several of the density parameters are given per hectare, whereas the resolution and the description of the study area are given in kilometers. (1) Some description of how many hectares the study area covers would be useful context. (2) More information on the study area and how the climate/human population/mosquito data relate to each other would be helpful. I suggest replacing/changing the map in Figure S2 to cover a wider area and have on it an outline of the municipality and the 13km2 area of the mosquito surveillance and a marker for the weather station, and including a scale bar.

Line 71. The suitability of the area for *Aedes albopictus* is given as 68%. What does this mean? E.g. is that 68% of the spatial area, or for 68% of the time? It sounds important so more explanation of what this value means would be useful.

Line 107. More description of the ovitrap data is needed. Are they *Aedes* specific data or mixed mosquito population? Are the ovitraps in similar locations to the BG sentinel 2 traps?

Line 376. Reference 30 is called a global model of arboviral transmission. I believe this reference is Americas and Europe only, not global. I suggest changing to one of the available global risk maps or rephrasing the sentence.

Line 365. I’m unsure about this sentence because you also have increased mortality of mosquitoes in the field compared to the lab.

Line 358 about the potential role of temporary tourist populations in arboviral transmission. Totally optional, but it might be interesting to comment on other high-tourist locations where outbreaks have been seen to highlight the risk to places similar to Chania (I’m thinking in particular of the dengue outbreak in Madeira 2012).

An optional suggestion it might be interesting to consider, is how many importations in an example cell would be needed for a ZIKV outbreak? There is zero risk here when one importation is considered, but for example, if a small group of infected travellers arrived on one importation date would that be enough to seed an outbreak?

Table S3 – what units are those rates?

Typesetting

Line 26 zika should be capitalised.

Line 99 Aedes should be italicised.

Map caption says Fig 3 should it be Fig 5? And Fig 4 to Fig 6.

In Line 361 and whole of that paragraph Aedes needs italicising.
