# Supplementary material for: Assessing risks of dengue, chikungunya and Zika transmission associated to Aedes albopictus in Chania, Greece, 2017–2018
Source: PLoS Negl Trop Dis. 2025 Dec 1;19(12):e0013785. doi: 10.1371/journal.pntd.0013785 (PMC12700377; doi:10.1371/journal.pntd.0013785)
Supplement: Attachment — Submitted filename: shadid25arboviralrisks-Response_to_reviewers.docx [file pntd.0013785.s005.docx]

**Response to reviewers**

**Reviewer #1:**

**Methods**

Clarity of objectives and hypothesis: The primary objective – to assess arboviral transmission risks in Chania – is clearly articulated. An implicit hypothesis is that there is a non-negligible risk of local transmission for dengue, chikungunya, and/or Zika following an imported case. This could be stated more explicitly.

We thank the reviewer for their appreciation of our work. Our objective was to quantify potential risks (if any) of local transmission for the three arboviral diseases. The non-negligible risks for dengue and chikungunya transmission and negligible risk for Zika are a result of our assessment. We now rephrased the study aim in the Introduction as follows:

Lines 86-91: " This study aims to assess and quantify potential epidemiological risks associated with dengue, chikungunya, and Zika transmission in Chania. We used mathematical modeling techniques to estimate the population density of *Ae. albopictus* mosquitoes, determine basic reproduction numbers for the three viruses, and assess outbreak probabilities, i.e. the risk that cases introduced (e.g., via travelling from endemic areas) will lead to sustained local transmission, as opposed to the virus fading out with few or no secondary infections.”

Parameterization: The model relies on temperature-dependent parameters. For chikungunya and Zika, temperature-independent epidemiological parameters were used. Given that chikungunya risk was found to be notably higher than dengue (whose model included temperature-dependent parameters), a sensitivity analysis on the key time-independent parameters for chikungunya is required to assess the robustness of this important finding and to understand how sensitive the higher chikungunya risk is to these fixed parameter values.

We agree with the reviewer on the importance of exploring the robustness of our results with respect to different parameterizations for dengue and chikungunya. We considered epidemiological parameters for *Ae. albopictus* to be constant with respect to temperature, in absence of published data on their temperature dependence. However, the experimental temperature for the measurement of such parameters is on the upper side of the temperature range observed in Chania (generally around 28°C), resulting in a possible overestimation of chikungunya transmissibility. Following the reviewer’s suggestion, we conducted a sensitivity analysis where we considered temperature-dependent functions of mosquito susceptibility, host susceptibility, and extrinsic incubation period for chikungunya, borrowed from the corresponding ones established for dengue, but rescaled in such a way to match the experimental value for chikungunya at the experimental temperature. If $f_{x}(T)$ is the temperature-dependent function observed for parameter $x$ for dengue, $G_{x}$ is the value of the constant chikungunya parameter and $T_{x}^{exp}$ is the temperature at which the experiment for $G_{x}$ was conducted, we considered a temperature-dependent function for chikungunya given by $g_{x}\left( T \right)= a\cdot f_{x}(T)$, where $a=\frac{f_{x}\left( T_{x}^{exp} \right)}{G_{x} =}$. We verified that the resulting values remained in physically plausible ranges (i.e., all values were non-negative and susceptibility parameters never exceeded 100%).

Re-running chikungunya simulations under these assumptions resulted in qualitatively similar results as in the main analysis (Figures S19-S21), although the transmissibility in the later part of the season decreased more briskly. This resulted in shorter season lengths and lower risks for chikungunya compared to the main analysis, but still larger corresponding estimates for dengue. This result should thus be attributed to the generally higher competence of *Aedes albopictus* in transmitting chikungunya compared to dengue [Gloria-Soria et al., Am J Trop Med Hyg 2020], rather than to the use of constant parameters with respect to temperature. We now provide this sensitivity analysis in a dedicated section of the Supplementary Material and mention the result in the Methods, Results, and Discussion:

Lines 169-170: “Temperature-dependent parameters for chikungunya, adapted from dengue, were also explored in a sensitivity analysis (see Supplementary Material).”

Lines 244-246: “Similar R_0_(t) estimates for chikungunya were obtained in a sensitivity analysis in which temperature-dependent parameters were used (see Supplementary Material).”

Lines 352-357: “Similarly to results previously reported for Northern Italy [28], chikungunya tended to have longer epidemic seasons and higher risks of outbreaks compared to dengue, due to the generally higher competence for chikungunya transmission [36]. This result was robust to using dengue-inspired temperature-dependent functional forms for epidemiological parameters of chikungunya rather than constant values from experimental studies (see Supplementary Material).”

Definition of "Widespread Outbreak" in TER: The TER calculation defines an outbreak as ≥10 infections. While any threshold is somewhat arbitrary, the rationale for this specific value should be discussed and the authors should briefly consider how varying this threshold might impact TER estimates and the comparison with IER.

We apologize for the lack of clarity. We agree that the term “widespread outbreak” is misleading and that the choice of 10 infections is somewhat arbitrary. This threshold was chosen according to the following reasoning: if the reproduction number is on average close to the epidemic threshold, stuttering transmission chains will require several generations before reaching 10 secondary infections, thereby representing a continuous alert to public health authorities over a breeding season; if the reproduction number is sufficiently larger than the epidemic threshold, the 10 secondary infections will be reached in a few generations, but due to the high reproduction number, transmission will self-sustain to produce many more cases, again representing a relevant public health threat. Thus, we have revised our definition of the 10 cases threshold as corresponding to “outbreaks of public health relevance” rather than “widespread outbreaks”.

The impact of varying the threshold on TER estimates was analyzed in depth in the study that introduced this measure [Kaye et al., Plos Comp Biol 2025]. The paper showed that the value of the TER is rather stable with respect to the threshold M at the beginning of the season (May-July) and tends to decrease with M late in the season (from September on; see Figure 5b from the paper reported below), mostly due to the fact that the end of the mosquito season limits the time available for further generations of infection.

This implies that the difference between TER and IER will be impacted mostly on the second half of the season, with the choice of a larger threshold amplifying the difference with IER.

We thank the reviewer for this important comment which we think allowed us to increase the clarity and scope of our results. We now included these considerations in the Discussion as follows:

Lines 404-410: “Our TER estimates are sensitive, especially in the second half of the mosquito season, to the choice of the number of infections for an outbreak to be considered relevant for public health [30]. Our choice of 10 infections or more includes either outbreaks with high transmissibility, or, given a generation time of two to three weeks for arboviral outbreaks [39,40], stuttering transmission chains over a significant portion of the breeding season; we contend that either of these possibilities are worthy of public health authorities being alerted to an ongoing outbreak.”

**Results**

Te Results section (particularly concerning Figure 3 and Figure 4) tends to be more descriptive of the figures' content rather than providing an immediate interpretation of what those descriptions signify. While the Discussion section later contextualizes these findings, weaving in some level of interpretation directly within the Results can indeed enhance clarity and demonstrate the added value of each figure more immediately

We have revised the text, moving substantial parts of the interpretations of results from the Discussion to the Results section, while keeping in the Discussion the contextualization of results and the assessment of the study limitations.

Figure 6: The purpose of the IER/TER comparison could be more clearly signposted. It appears to serve a dual role: 1) to demonstrate that differences exist between the metrics, and 2) to explore the determinants of these differences.

Perhaps structuring the paragraph in the Results more explicitly. For instance, you could first discuss the direct comparison of IER and TER (from the box plots), highlighting the general patterns. Then you could introduce the regression analysis specifically as a means to understand why these differences occur and what factors influence them.

We thank the reviewer for this suggestion. We revised the paragraph in the Results accordingly.

**Conclusions**

There are several additional limitations or areas for more detailed discussion:
- The authors should clarify whether any routine or reactive vector control measures were ongoing in Chania during the 2017-2018 study period. Such activities could significantly influence local mosquito densities and affect model calibration and risk assessments, acting as an unmeasured confounder.

- While the study used meteorological data, mosquito development and survival are highly influenced by microclimatic conditions (e.g., in shaded areas, breeding sites).

- human behavioral factors e.g., human mobility, time spent outdoors, use of repellents, or socio-economic factors influencing housing conditions

We agree with the reviewer about these important points.

Unfortunately, we have no information on the occurrence of vector control measures in the area of mosquito collection that may have affected calibration; however, we point out that the mosquito trapping area was a rural one, therefore we believe it is unlikely that it was subject to any intervention. Vector control measures might have been taken within the urban area of Chania, thereby altering the actual modelled risks. Because our simulations do not take into account potential interventions, the estimates must be considered as potential risks in absence of interventions. We also acknowledge that microclimatic and environmental conditions, including the availability of food, shaded areas, breeding sites, and the abundance of predators can all affect development and survival rates of different mosquito developmental stages; however, these data are difficult to collect and their mechanistic impact is poorly quantified. In our model, we summarize the environmental suitability within a single factor, identified in the larval overcrowding rate that is estimated separately for each trap. Ignoring the variability of environmental suitability in the territory of Chania is a simplification that we have now explicitly acknowledged in the Discussion. Similarly, the potential variability of exposure risks associated to human behavioral factors needed to be better acknowledged and we have now done so. We are grateful to the reviewer for these suggestions which allowed us to improve the contextualization of our results.

Lines 398-404: “The study did not include possible spatial heterogeneities in microenvironmental conditions across the town, including the availability of food, shaded areas, breeding sites, and the abundance of predators, which can affect the development and survival rates across different mosquito developmental stages; similarly, the availability of temperature data from a single weather station for the whole municipality did not enable the explicit consideration of local microclimatic conditions that may influence mosquito proliferation and arboviral risks.”

Lines 434-442: “We did not account for vector control measures in the area of mosquito collection that may have affected calibration of the model; however, we point out that the mosquito trapping area was a rural one, therefore we believe it is unlikely that it was subject to any intervention. Vector control measures may have been implemented within the urban area of Chania, thereby altering the outbreak risk. Because our simulations do not explicitly account for interventions, the estimates must be considered as potential risks in absence of interventions. Finally, the impact of human behavioral factors was not considered: protective actions such as the use of repellents, time spent outdoors and socio-economic characteristics (e.g., housing conditions) should also be investigated to improve the accuracy of risk assessments.”

**Editorial and Data Presentation Modifications**

Introduction:

Line 61-62: "Surveillance studies have been conducted in..." Please clarify if these refer to mosquito surveillance, disease surveillance, or both. A brief elaboration on the nature of these cited surveillance studies (e.g., "focused on vector distribution and abundance") would add context.

We apologize for the lack of clarity. We have now rephrased the text to indicate more clearly that the cited studies addressed both epidemiological and entomological surveillance and provided evidence of the vector’s distribution and abundance. The revised text reads as follows:

Lines 60-64: “This mosquito vector has now established populations across much of southern and central Europe, displaying an ability to adapt to temperate climates [6]. This has been highlighted by several epidemiological and entomological surveillance studies focused on its distribution and abundance, conducted in Switzerland [7], Spain [8], Germany [9], Italy [10], and Greece [11].”

Line 63-64: The statement "for which viral transmissibility is directly proportional to the mosquito population density" is a slight oversimplification. Consider rephrasing to something like "is strongly influenced by" or "generally increases with" mosquito population density.

We agree and have rephrased accordingly: “viral transmissibility generally increases with the mosquito population density”.

The labeling of Figures in the main text captions appears incorrect from Figure 4 onwards in the submitted manuscript

We thank the reviewer for spotting this mistake. We have corrected the labeling accordingly.

Figure 5 : For panels showing generally very low risk (eg Dengue-TER) it would be helpful to include the boundary of the municipality of Chania as a light grey overlay or base map. This would provide better spatial context and make it easier to discern the assessed area, especially where many cells are blank or light-colored.

We agree with the reviewer that the representation of the spatial result needed improvement. However, the administrative borders of the municipality of Chania, according to the latest Greek administrative reform, include a much larger area than the residential area displayed in Figure 5, and therefore its inclusion would not be informative. The geographical extent of the Chania municipality is shown in the revised version of Figure S2 which now includes the position of Crete within Greece, of the Chania municipality administrative borders within Crete, and of the mosquito trapping areas, weather station, and modelled residential population as well as a scale bar and a North arrow.

In order to further help the reader understand the overlap between the residential and modelled area, we added a dark outline to modelled cells in Figures 5, S8, S9, S13 and S14, allowing to better differentiate non-modelled areas and areas with very low risk. We thank the reviewer for this suggestion.

Figure 6 : The notations "Changes in 1 unit of host density corresponds to increase of X in the difference" directly on the right panels, while informative, contribute to visual clutter. Consider moving this quantitative detail to the figure caption or the main text

We thank the reviewer for this suggestion; we moved this information in the Results text.

**Summary and General Comments**

This manuscript investigates the transmission risk of dengue, chikungunya, and Zika viruses by Aedes albopictus in Chania, Greece, a region with established vector presence but no history of local arboviral outbreaks. The study is timely, given the increasing establishment of Ae. albopictus in Europe and the potential for virus importation via international travel, particularly to tourist hotspots like Chania.

Strengths of the study include its application of a mathematical model calibrated with local, multi-year entomological surveillance data, its assessment of multiple arboviruses, and its insightful comparison of two different risk metrics (IER and TER). The finding of a non-negligible risk, particularly for chikungunya, and the spatial identification of higher-risk areas (peripheral, less densely populated zones) provide importnat information for public health authorities. The discussion on the limitations of the simpler IER metric is a valuable methodological contribution.

The primary weaknesses lie in the limitations imposed by some methodological assumptions that need further justification or exploration as outlined above.

We thank the reviewer for their appreciation of our work and for helping us improving the robustness and clarity of the manuscript. We hope that the applied changes help overcome the weakness pointed out by the reviewer.

**Reviewer #2**

**Results**

The figures and results are clearly communicated, and the analysis plan matched the final results. However, no results of the raw capture data, which is an important part of the methodology is presented in the results.

We thank the reviewer for this suggestion. We have now moved to the main results the supplementary figure showing the model fit to the capture data (Figure 1). We also added the following information to the Results:

Lines 224-233: “The entomological surveillance collected a total of 730 adult females of *Ae. albopictus* across the eight traps between May 1 and December 31, 2017, and 735 from April 1 to December 31, 2018. The model reproduced the general seasonal trends in the average mosquito captures (Figure 1A-B), as well as capture data from the individual traps (see Supplementary Material). The trap with the highest abundance collected a total of 268 adult females in 2017 and 453 in 2018, with peaks of respectively 34 and 58 captures in August. The resulting estimated mosquito abundance shows substantial variations in mosquito densities between the two seasons, with a mean peak of 459 (95% Credible Interval, CrI: 424-508) females per hectare in 2017 and 757 (95% CrI: 728-785) in 2018 (Figure 1C-D), occurring in both years during the second half of September.”

**Conclusions**

There is discussion of the results, and limitations of the methods used. There is also public health relevance mentioned, although a slight fleshing out of this could be beneficial

We thank the reviewer for their appreciation of our work. Following comments from all reviewers, we now substantially expanded the text to better define the scope, public health relevance, and limitations of our study. We hope that the revised version now more clearly conveys the public health implications of our findings

**Summary and General Comments**

This is a publication examining the risk of transmission of three arboviruses (dengue, Zika, and Chikungunya, in Chania, Greece, using mosquito surveillance, population, and climate data in a model to calculate the probability of local transmission. They found risks of outbreaks of chikungunya and dengue in this context and discuss some implications. This is a timely topic and requires only minor changes.

1. On lines 69-71, introducing the concept of suitability factor, but now defining it may introduce confusion. Either expand or remove the suitability factor percentage and simplify.

We apologize for the lack of clarity. The reported 68% suitability for Chania was reported in the context of Tagaris et al. (in Perspectives on Atmospheric Sciences, 2017) and reflected a model-derived index of climatic favorability for *Ae. albopictus*. Generated through the integration of meteorological observations and dynamically downscaled climate projections, this index (on a 0–100% scale) assesses the alignment of local environmental conditions with the species' ecological requirements for establishment and persistence. Thus, a value of 68% indicated a moderately high degree of climatic concordance. This theoretical assessment was supported by empirical field risk assessments based on egg density data [Bellini et al., Vect Biol J, 2016; Badieritakis, Paras Res, 2018]. Following the reviewer’s suggestions, to avoid confusion we simplified the text removing the quantitative value and referring to a qualitative assessment of the climatic suitability:

Lines 75-78: “A previous study found a moderately high climatic suitability in the region for the establishment and proliferation of the mosquito, enabling potential arboviral transmission following the importation of an infectious case from endemic areas [18].”

2. Optional: Mention of specific outbreaks of VBDs (chikungunya in Italy perhaps) with specific years and numbers in the introduction to underscore the importance of this work.

We thank the reviewer for this suggestion. We now added the following paragraph to the Introduction:

Lines 70-72: “Only in 2024, local dengue transmission totaled 83 cases in France and 213 cases in Italy; in 2025, as of October 1, 637 locally transmitted cases of chikungunya were recorded in France and 323 in Italy [14,16].”

3. Make sure Aedes is always in italics.

We thank the reviewer for the careful reading. We corrected formatting errors accordingly.

4. There are no direct results from the mosquito trapping reported (outside of the abstract). Actual numbers of trapped mosquitoes and species would benefit the paper, and not only the modelled mosquito densities, especially when the importance of such trapping and surveillance is emphasized in the discussion.

We agree with the reviewer, and we modified the manuscript accordingly (see comment above).

5. An expansion in the discussion of previous reports of imported cases of the mentioned arboviruses would also be interesting.

We included the following sentence in the Discussion:

Lines 444-446: “Based on the Greek National Public Health Organism, in recent years, only imported cases of these diseases have been recorded, in limited numbers, among travelers arriving from endemic countries abroad [42,43].”

6. A slight expansion in the discussion of the implications of these findings and diving deeper into prevention would also be a benefit. How effective would public information campaigns about standing water, or mosquito bite prevention in summer months be? Should insecticide campaigns be governmental, local, personal?

We thank the reviewer for their suggestion. We expanded the Discussion with results from previous research suggesting the cost-effectiveness of larviciding in small towns (usually conducted by municipal authorities) and cited other studies about public information campaigns:

Lines 465-473: “The existence of non-negligible arboviral outbreak risks suggests the importance of integrated vector management to reduce the risks of onward transmission, especially between mid-summer and early autumn. Previous studies demonstrated the cost-effectiveness of undertaking routine larvicide treatment in public spaces for medium-to-small municipalities [53], and the effectiveness of door-to-door interventions with public awareness campaigns for breeding site removal [54,55]. Enhancing surveillance systems to facilitate the early detection of local arboviral transmission can be critical to reduce the burden of potential ensuing outbreaks [40, 56].”

**Reviewer #3**

Summary

Dear editors,

This review is for the article “Assessing risks of dengue, chikungunya and Zika transmission associated to Aedes albopictus in Chania, Greece, 2017-2018.” The paper is original and interesting to read. I have written my review comments below, I hope the authors find them helpful. My main comment is that the methods section needs expanding because it is often unclear what was performed without going back and forth to the Supplementary Material, and so I believe more methodological detail is needed in the main text.

Overall, the content of this paper meets the requirements from the journal: it is of interest to researchers and practitioners outside the field as well as in the field of NTDs. I have split my comments into clarifications that are necessary and also more minor suggestions which I hope the authors find helpful. Thank you to the authors for writing this interesting paper.

We thank the reviewer for their appreciation of our work and for the useful comments. We provide below a point-to-point response to each.

Major points

Line 135. The free model parameters include the larval-overcrowding and the capture rate for the adult females. How was the larval-overcrowding calibrated? Was this using the ovitrap data? This is unclear and more explanation would be helpful.

We apologize for the lack of clarity. The larval-overcrowding and capture rate were both calibrated through MCMC by fitting model estimates to adult female mosquito capture data over time. The ovitrap collection was part of the entomological surveillance but no ovitrap data has been used for this work. For the record, ovitrap surveillance involved thirty traps placed in rural villages and twenty-one in surrounding agricultural areas to monitor oviposition activity. In contrast, BG-Sentinel traps were deployed exclusively in rural areas to target host-seeking adults. All ovitrap data were collected following the standard operational procedures for field management outlined by Bellini et al. (2020) and are fully available in Stefopoulou et al. (2022). To avoid further confusion to the reader, we decided to discard any reference to ovitraps from the manuscript. We have made the calibration more explicit in the text:

Lines 139-142: “Free model parameters were the year-specific larval overcrowding [30] and the effectiveness of traps in capturing adult females [28, 31], and were calibrated against adult female mosquito capture data throughout the season, using a Markov Chain Monte Carlo (MCMC) approach.”

Line 147. “considering mosquito populations associated to the trap with highest abundance over each year”. Does this mean that data from only one of the traps was used? It is unclear why this was done, please can you expand on the reasoning. How do the results change if the mosquito population is averaged over the traps instead? If I’ve understood correctly what data was used here, then this needs to be talked about in the discussion as a potential introduction of bias.

We apologize for the lack of clarity and we thank the reviewer for the opportunity to improve the manuscript on this point. The mosquito population model was calibrated for each trap and year, as reported in the Supplementary Materials (Figure S3 and S4); in the main text we also show the correspondence between observed and modelled captures averaged across the 8 traps. As the reviewer correctly points out, potential epidemiological risks in Chania were then estimated using the highest mosquito abundance estimated across traps in each year. To justify this choice, we first note that traps were located in rural areas and resulted in a heterogeneous yield, both across traps in the same year, and for a given trap over the two years considered (Figure S3 and S4). This outcome is likely associated with heterogeneities in microclimatic and microenvironmental conditions affecting mosquito proliferation and survival as well as capture dynamics (e.g., traps placed in areas with stronger winds or less shadow might have been less attractive). We preferred to avoid averaging abundance estimates across traps since several traps had very low yield and this may result in more biased estimates due to stochastic noise. Residential areas tend to be more favorable environments for *Ae. albopictus* than rural ones, therefore we chose traps with the maximum estimated mosquito abundance as the best proxy for the residential area of Chania. In summary, the environmental conditions in the Chania area were compatible with *at least* the abundance estimated from those traps, therefore we consider potential risks of arboviral to be associated to these values.

We now make this more explicit in the Methods and we discussed our assumption and the associated limitation in the Discussion.

Lines 162-164: “For the mosquito population, $N_{V}$, we used estimates associated with the trap with highest estimated abundance over each year.”

Lines 391-398: “In particular, we used the maximum mosquito abundance estimated across traps for each year, which does not necessarily reflect the mosquito population within the residential area of the town. The choice of the maximum estimated mosquito abundance seemed the most appropriate given the available data for two reasons: first, urban areas are known to be more favorable for *Ae. albopictus* proliferation than rural areas; second, it is more risk averse to consider the worst observed conditions to avoid underestimating potential risks. However, this choice needs to be considered when interpreting our results.”

Line 145. Is R0(t) the R0 or is it Rt? Please clarify this in the text.

We estimated the basic reproduction number R0 at different times, hence the notation R0(t). The time-dependence derives from the seasonal vector abundance and from temperature- (and therefore time-) dependent parameters. We have now clarified this in the Methods:

Lines 149-151: “To estimate the risk of chikungunya, dengue and Zika outbreaks in Chania, we computed the basic reproduction numbers R0(t), which vary over time due to seasonal changes in vector abundance and in temperature-dependent parameters.”

This work originally looked like a spatial model to me. Whereas I now see that the model was run independently for each of the 147 cells with no modelling of correlation across space. This is fine, but I think the distinction needs to be made clearer from the beginning of the article. Minor suggestion on this point - it could be useful to give an overall outbreak risk value for the whole municipality, assuming a constant averaged human population density first, and then describe the cell-specific results. Or have a graph of risk versus human population perhaps.

We apologize for the lack of clarity. We have now clarified that the risk is computed independently on the 147 cells with no modelling of spatial correlation and the only difference across cells being the human population density as follows:

Lines 170-172: “We computed R_0_(t) separately for each of the 147 cells of the residential area of Chania using the corresponding human population density (i.e., spatial correlation across cells was neglected).”

We additionally provided in the Supplementary Material new figures (Figure S11 and S12) where we show the negative correlation between the human population densities of each cell and both the average IER and season duration. Note that the relationship is not identical in different years and that it cannot be obviously reconducted to a simple analytical function.

Similar to the point above, the only difference between the cells is the human population density. The mosquito population and climate are constant across the whole area and any spatial difference in R0 and outbreak risk is wholly dependent on the human population density. (1) Is it possible there could be large spatial differences in the mosquito population density across the municipality? It seems from the supplementary figures that even across the 8 traps there was some heterogeneity. Some more discussion is needed around how spatial differences in mosquito populations would impact the results. Could areas of higher human population density sustain a larger mosquito population carrying capacity?

The reviewer is correct in their interpretation that the cells only differ by human population density. Spatial temperature data were not available and, as mentioned above, mosquito abundance data referred to an area that did not correspond to the modelled residential area, thereby providing no information on the possible spatial variability within the town of Chania. While *Aedes albopictus* does prefer urban environments to rural ones, the effect of human population density on mosquito abundance within a municipality residential area is more difficult to assess. We expect that increasing human population densities may favor the mosquito abundance up to a given point; however, at some point a high population density needs to be supported by dense multi-store buildings, which saturate the availability of breeding sites per inhabitant (such as catch-basins), and leave little space for vegetation, which is necessary for shelter and nutriment, thereby eventually reducing the local mosquito carrying capacity. Previous studies comparing estimates from different municipalities [Guzzetta et al., Plos NTD, 2016] showed non-significant relationships between reproduction numbers and human population densities for a range of values comparable to the Chania residential area. We now expanded the Discussion to better acknowledge the model limitations on this issue.

Lines 398-404: The study did not include possible spatial heterogeneities in microenvironmental conditions across the town, including the availability of food, shaded areas, breeding sites, and the abundance of predators, which can affect the development and survival rates across different mosquito developmental stages; similarly, the availability of temperature data from a single weather station for the whole municipality did not enable the explicit consideration of local microclimatic conditions that may influence mosquito proliferation and arboviral risks.

(2) Because so much of the results focusses on the differences in risk between the cells, more information/equations on how human population density is used to calculate the R0 and risk is needed in the main methods text.

Following the reviewer’s suggestion, we now moved the equation for R_0_ and the IER in the Methods description in the main text.

In the Supplement it says that for dengue, it was assumed that only individuals displaying symptoms contribute to virus transmission. What is the justification for this?

We apologize for the lack of clarity. The role of asymptomatic individuals in transmission remains debated, with evidence from cohort studies suggesting that asymptomatic dengue infections are characterized by lower viral titers and distinct viremia kinetics compared to symptomatic cases, possibly resulting in lower infectiousness (Matangkasombut et al., International Journal of Infectious Diseases, 2020). Modeling studies have adopted different assumptions about the relative transmissibility of symptomatic and asymptomatic cases. A modeling study by Ferguson et al (Science 2016) assumed in the main analysis that symptomatic infections are twice as infectious as asymptomatic infections. This results in a scaling factor for transmissibility that ranges from 0.5 to 1, depending on the proportion of symptomatic individuals. In our approach, we adopt a similar rationale, acknowledging the potentially higher contribution of symptomatic individuals to transmission. We defined a factor p that ranges from 0.25 to 1 to be multiplied by R0, reflecting different assumptions about the relative transmissibility of symptomatic and asymptomatic individuals. This formulation captures scenarios where symptomatic individuals represent a smaller proportion of infections - such as in endemic countries, where they typically account for around 20% - yet contribute more to transmission than asymptomatic cases, as well as others where symptomatic individuals are the majority (such as in a dengue outbreak in the Lombardy region, Italy; see Rovida et al, Travel Medicine and Infectious Disease, 2025) or where all individuals, asymptomatic and symptomatic, contribute equally to the transmission (p=1). It is worth noting that the factor p, under the assumption that asymptomatic individuals do not transmit, coincides with the fraction of symptomatic individuals. However, p should not be interpreted strictly as the fraction of symptomatic individuals, but rather as a proxy for the overall contribution of all infected individuals (asymptomatic and symptomatic) in a context where symptomatic individuals may be more infectious. We now make this explicit in the Supplementary Material.

Table S4 has mosquito susceptibility for ZIKV as a very low maximum ~10% whereas 70-100% for CHIKV which is likely why the authors see no risk for ZIKV outbreaks. Since it has such a big impact on the results, I think more discussion on these values would be useful. i.e. are the referenced % values representative of the literature? Is there any chance the ZIKV value could be higher and therefore the risk underestimated?

We thank the reviewer for rising this important point. Because vector competence depends substantially on the mosquito strain considered, we considered estimates for mosquito populations coming from geographical areas close to the study area. In particular, ZIKV competence estimates derive from an experimental laboratory infection study on Italian mosquitoes [Di Luca et al., Eurosurv 2016], whereas the high range for CHIKV estimates derives from studies on Italian and French mosquitoes [Fortuna et al, Eurosurv 2018; Vazeille et al, Acta Tropica, 2018]. To the best of our knowledge, there is currently no evidence of higher mosquito susceptibility for ZIKV in other mosquito populations from Europe. However, this may change in the future due to viral evolution and adaptation: it is therefore important to continuously monitor the competence of mosquito populations for different arboviruses. We now expanded our Discussion to include these considerations:

Lines 461-465: “In contrast, as previously found for temperate areas in Italy, the risk of Zika outbreaks was estimated to be negligible due to the poor competence of European *Ae. albopictus* strains [52]. However, this may change in the future following viral evolution and adaptation: it is therefore important to monitor changes in the competence of mosquito populations for different arboviruses over time.”

The data availability statement suggests that all data is available, however the mosquito capture data is only included in a figure and therefore not practically available. For reproducibility, this article would benefit from source code being made available for example through a GitHub repository, and data similarly made available externally or at least given in tables. If the mosquito data can’t be made available (I appreciate this is often not possible) then the data availability statement needs to be updated to express under what circumstances researchers could request access and from where.

We apologize for the oversight. We have now included the mosquito capture and temperature data used in the study as supplementary files and amended the statement accordingly.

Minor points

Several of the density parameters are given per hectare, whereas the resolution and the description of the study area are given in kilometers. (1) Some description of how many hectares the study area covers would be useful context.

We apologize for the lack of clarity. Each cell represents a square of area 6.25 hectares, therefore the total study area is given by 6.25*147 = 918.75 hectares. We now report this information in the Methods.

(2) More information on the study area and how the climate/human population/mosquito data relate to each other would be helpful. I suggest replacing/changing the map in Figure S2 to cover a wider area and have on it an outline of the municipality and the 13km2 area of the mosquito surveillance and a marker for the weather station, and including a scale bar.

We now updated the map in Figure S2 to show the position of Crete within Greece, of the Chania municipality administrative borders within Crete, and of the mosquito trapping areas, weather station and modelled residential areas. We included a scale bar and a North arrow. We thank the reviewer for this useful suggestion.

Line 71. The suitability of the area for Aedes albopictus is given as 68%. What does this mean? E.g. is that 68% of the spatial area, or for 68% of the time? It sounds important so more explanation of what this value means would be useful.

We apologize for the lack of clarity. The reported 68% suitability for Chania was reported in the context of Tagaris et al. (in Perspectives on Atmospheric Sciences, 2017) and reflected a model-derived index of climatic favorability for *Ae. albopictus*. Generated through the integration of meteorological observations and dynamically downscaled climate projections, this index (on a 0–100% scale) assesses the alignment of local environmental conditions with the species' ecological requirements for establishment and persistence. Thus, a value of 68% indicates a moderately high degree of climatic concordance. This theoretical assessment was supported by empirical field risk assessments based on egg density data [Bellini et al., Vect Biol J, 2016; Badieritakis, Paras Res, 2018]. Following the reviewer’s suggestions, to avoid confusion we simplified the text removing the quantitative value and referring to a qualitative assessment of the climatic suitability:

Lines 75-78: “A previous study found a moderately high climatic suitability in the region for the establishment and proliferation of the mosquito, enabling potential arboviral transmission following the importation of an infectious case from endemic areas [18].”

Line 107. More description of the ovitrap data is needed. Are they Aedes specific data or mixed mosquito population? Are the ovitraps in similar locations to the BG sentinel 2 traps?

We apologize for the lack of clarity. The ovitrap collection was part of the entomological surveillance but no ovitrap data has been used for this work. For the record, ovitrap surveillance involved thirty traps placed in rural villages and twenty-one in surrounding agricultural areas to monitor oviposition activity. In contrast, BG-Sentinel traps were deployed exclusively in rural areas to target host-seeking adults. All ovitrap data were collected following the standard operational procedures for field management outlined by Bellini et al. (2020) and are fully available in Stefopoulou et al. (2022). To avoid further confusion to the reader, we decided to discard any reference to ovitraps from the manuscript.

Line 376. Reference 30 is called a global model of arboviral transmission. I believe this reference is Americas and Europe only, not global. I suggest changing to one of the available global risk maps or rephrasing the sentence.

We apologize for the oversight. The reviewer is correct, as the reference was for the Americas and Europe only. We now added a reference to two global risk maps [Messina et al., Nat Microbiol, 2019; Lim et al., Nat Comm, 2025] and revised the sentence as follows:

Lines 459-461: “This result obtained from entomological empirical data corroborates previous predictions obtained from global [50,51] and continental [31] models of arboviral transmission.”

Line 365. I’m unsure about this sentence because you also have increased mortality of mosquitoes in the field compared to the lab.

We agree. We rephrased as follows:

Lines 444-450: “Based on the Greek National Public Health Organism, in recent years, only imported cases of these diseases have been recorded, in limited numbers, among travelers arriving from endemic countries abroad [42,43]. Laboratory experiments have also demonstrated the vector competence of *Aedes* populations in the region, even when females were offered moderately low initial virus titers [44], suggesting that vector competence in natural field conditions could be higher, potentially amplifying transmission risks; on the other hand, field conditions may lead to higher mosquito mortality, thereby decreasing transmission risks”.

Line 358 about the potential role of temporary tourist populations in arboviral transmission. Totally optional, but it might be interesting to comment on other high-tourist locations where outbreaks have been seen to highlight the risk to places similar to Chania (I’m thinking in particular of the dengue outbreak in Madeira 2012).

Thank you for the suggestion. We added a reference to the chikungunya outbreak which spread in the seaside municipality of Anzio, in central Italy, since late May / early June 2017 [Manica et al., Eurosurv, 2017]. The outbreak caused over 200 locally transmitted cases in Anzio and seeded a large number of smaller outbreaks in Rome and in other neighboring municipalities within the Lazio region (totalling another 200 cases overall). Another large outbreak of approximately 100 cases was seeded from Anzio to the municipality of Guardavalle Marina, in Calabria Region, about 600 km away. A complete analysis of spatial dynamics of transmission for the Lazio cases is reported in [Guzzetta et al., BMC Med 2020]. We now added this text to the Discussion.

Lines 426-432: “In 2017, a chikungunya outbreak started around early June in Anzio [38] in the Lazio region of Italy, a popular seaside destination for inhabitants of the region; in addition to over 200 locally transmitted cases in Anzio, the outbreak seeded a number of smaller self-contained outbreaks in the surrounding areas, including the municipality of Rome (adding up to approximately 200 further cases) [40], and a secondary large outbreak of about 100 cases in the municipality of Guardavalle Marina, 600 km from Anzio.”

An optional suggestion it might be interesting to consider, is how many importations in an example cell would be needed for a ZIKV outbreak? There is zero risk here when one importation is considered, but for example, if a small group of infected travellers arrived on one importation date would that be enough to seed an outbreak?

We thank the reviewer for pointing this out this interesting point. Unfortunately, the computation cannot be addressed using the IER, as it is an analytically derived quantity that is identically zero when R0 is below 1, regardless of the number of importations [Kaye et al., J Theor Biol 2022; Lloyd et al., J Royal Soc Interface, 2007]. A scenario with multiple importations could be explored using the TER, however the measure represents the probability of observing at least 10 infections with a single introduced case. Given the very low estimated values of R_0_ for Zika throughout the two seasons, the need to redefine the TER with multiple introductions and the computational intensity of such measure, we preferred to not implement this suggestion.

Table S3 – what units are those rates?

We apologize for the lack of clarity. We have now added the unit of measures in Table S3. The larval overcrowding is adimensional and represents how many additional larvae in the modeled population will cause a 100% increase in larval mortality with respect to the base value m_L. The capture rate is measured in days^-1^ and represents the proportion of existing mosquitoes in the modelled population that are captured by a given trap in 24 hours.

Typesetting

Line 26 zika should be capitalised.

Line 99 Aedes should be italicised.

Map caption says Fig 3 should it be Fig 5? And Fig 4 to Fig 6.

In Line 361 and whole of that paragraph Aedes needs italicising.

Corrected. We thank the reviewer for the careful reading.

**Editorial requests**

1) Please ensure that the CRediT author contributions listed for every co-author are completed accurately and in full.
At this stage, the following Authors/Authors require contributions: Shahid Nadim Sk, Francesco Menegale, Mattia Manica, Alexander R Kaye, Georgios Balatsos, Marina Bisia, Verena Pichler, Piero Poletti, Stefano Merler, Alessandra Della Torre, Robin N Thompson, Antonios Michaelakis, and Giorgio Guzzetta. Please ensure that the full contributions of each author are acknowledged in the "Add/Edit/Remove Authors" section of our submission form.
The list of CRediT author contributions may be found here: <https://journals.plos.org/plosntds/s/authorship#loc-author-contributions>

Done.

2) Please upload all main figures as separate Figure files in .tif or .eps format. For more information about how to convert and format your figure files please see our guidelines: 
<https://journals.plos.org/plosntds/s/figures>

Done.

3) Please ensure that all Figure files have corresponding citations and legends within the manuscript. Currently, Figure 2 in your submission file inventory does not have an in-text citation. Please include the in-text citation of the figure.

Done. We apologize for the oversight.

4) Please ensure that the figures are labeled correctly in a numerical order in the manuscript.

Done. We apologize for the oversight.

5) We have noticed that you have uploaded Supporting Information files, but you have not included a list of legends. Please add a full list of legends for your Supporting Information files after the references list.

Done.

6) Some material included in your submission may be copyrighted. According to PLOSu2019s copyright policy, authors who use figures or other material (e.g., graphics, clipart, maps) from another author or copyright holder must demonstrate or obtain permission to publish this material under the Creative Commons Attribution 4.0 International (CC BY 4.0) License used by PLOS journals. Please closely review the details of PLOSu2019s copyright requirements here: PLOS Licenses and Copyright. If you need to request permissions from a copyright holder, you may use PLOS's Copyright Content Permission form.
Please respond directly to this email and provide any known details concerning your material's license terms and permissions required for reuse, even if you have not yet obtained copyright permissions or are unsure of your material's copyright compatibility. Once you have responded and addressed all other outstanding technical requirements, you may resubmit your manuscript within Editorial Manager. 
Potential Copyright Issues:
i) Figures 5, S2, S7, S8, S9, S11, and S12. Please (a) provide a direct link to the base layer of the map (i.e., the country or region border shape) and ensure this is also included in the figure legend; and (b) provide a link to the terms of use / license information for the base layer image or shapefile. We cannot publish proprietary or copyrighted maps (e.g. Google Maps, Mapquest) and the terms of use for your map base layer must be compatible with our CC BY 4.0 license.
Note: if you created the map in a software program like R or ArcGIS, please locate and indicate the source of the basemap shapefile onto which data has been plotted.
If your map was obtained from a copyrighted source please amend the figure so that the base map used is from an openly available source. Alternatively, please provide explicit written permission from the copyright holder granting you the right to publish the material under our CC BY 4.0 license.
If you are unsure whether you can use a map or not, please do reach out and we will be able to help you. The following websites are good examples of where you can source open access or public domain maps:
* U.S. Geological Survey (USGS) - All maps are in the public domain. ([http://www.usgs.gov](http://www.usgs.gov/))
* PlaniGlobe - All maps are published under a Creative Commons license so please cite u201cPlaniGlobe, [http://www.planiglobe.com](http://www.planiglobe.com/), CC BY 2.0u201d in the image credit after the caption. (<http://www.planiglobe.com/?lang=enl>)
* Natural Earth - All maps are public domain. (<http://www.naturalearthdata.com/about/terms-of-use/>).

We apologize for the oversight. We are now including background maps from OpenStreetMap (<https://www.openstreetmap.org>) for Figures 5, S7, S8, S9, S13, and S14. OpenStreetMap data are licensed under Creative Commons Attribution-ShareAlike 2.0 license (https://www.openstreetmap.org/copyright/en) that allows, under appropriate crediting of OpenStreetMap, to:

1. Share - copy and redistribute the material in any medium or format for any purpose, even commercially.
2. Adapt - remix, transform, and build upon the material for any purpose, even commercially.

OpenStreetMap website states that “You are free to copy, distribute, transmit and adapt our data, as long as you credit OpenStreetMap and its contributors. If you alter or build upon our data, you may distribute the result only under the same license”. We have added the following statement to the captions of Figures 5, S7, S8, S9, S13, and S14:

“Maps were created using QGIS software version 3.30.2. Background map layer was obtained from OpenStreetMap (https://www.openstreetmap.org) and is made available under the Open Database License (http://opendatacommons.org/licenses/odbl/1.0/). Any rights in individual contents of the database are licensed under the Database Contents License (http://opendatacommons.org/licenses/dbcl/1.0/).”

Figure S2 was created using ArcGIS Pro (3.0.0). We have added the following statement to the caption:

“Background map layer was created with ArcGIS Pro (3.0.0), using the World Topographic Map available from Esri (Marathon Data, GR) under Esri Master License Agreement (https://www.arcgis.com/home/item.html?id=30e5fe3149c34df1ba922e6f5bbf808f). The administrative boundaries for the municipality of Chania were retrieved from [5], using functions available in the ggmap package for R version 4.0.0 [6], together with a private API key. ”

7) Please amend your detailed Financial Disclosure statement. This is published with the article. It must therefore be completed in full sentences and contain the exact wording you wish to be published.
1) State the initials, alongside each funding source, of each author to receive each grant. For example: "This work was supported by the National Institutes of Health (####### to AM; ###### to CJ) and the National Science Foundation (###### to AM)."
2) State what role the funders took in the study. If the funders had no role in your study, please state: "The funders had no role in study design, data collection and analysis, decision to publish, or preparation of the manuscript."
3) If any authors received a salary from any of your funders, please state which authors and which funders.

Done.

8) Thank you for stating "All the authors declare that they have no conflicts of interest." Please modify your Competing Interest statement on the submission form to the standard "The authors have declared that no competing interests exist."

Done.
